# Turning on the Heat: Ecological Response to Simulated Warming in the Sea

**DOI:** 10.1371/journal.pone.0016050

**Published:** 2011-01-14

**Authors:** Dan A. Smale, Thomas Wernberg, Lloyd S. Peck, David K. A. Barnes

**Affiliations:** 1 School of Plant Biology, Oceans Institute, University of Western Australia, Perth, Western Australia, Australia; 2 Australian Institute of Marine Science, Oceans Institute, University of Western Australia, Perth, Western Australia, Australia; 3 British Antarctic Survey, Natural Environment Research Council, Cambridge, United Kingdom; University of Hull, United Kingdom

## Abstract

Significant warming has been observed in every ocean, yet our ability to predict the consequences of oceanic warming on marine biodiversity remains poor. Experiments have been severely limited because, until now, it has not been possible to manipulate seawater temperature in a consistent manner across a range of marine habitats. We constructed a “hot-plate” system to directly examine ecological responses to elevated seawater temperature in a subtidal marine system. The substratum available for colonisation and overlying seawater boundary layer were warmed for 36 days, which resulted in greater biomass of marine organisms and a doubling of space coverage by a dominant colonial ascidian. The “hot-plate” system will facilitate complex manipulations of temperature and multiple stressors in the field to provide valuable information on the response of individuals, populations and communities to environmental change in any aquatic habitat.

## Introduction

A multitude of biologically significant environmental changes are projected to occur as a consequence of anthropogenic climate change [1]. Increasing temperature is arguably the most important change, as temperature influences physiological and ecological processes across biological scales, from genes to ecosystems. Current knowledge of observed and expected biological changes – and the ecophysiological mechanisms that drive them – on land far exceeds that from ocean systems, largely because of the costly, logistically-challenging nature of marine research and the inaccessibility of aquatic habitats [2]. For example, unlike in terrestrial habitats, where cloches have been used to experimentally elevate temperature over short to medium timescales to investigate community-level responses to simulated global warming [3], [4], there have been few controlled manipulations of temperature in the sea.

Although it is logistically difficult to manipulate seawater temperature for ecological field experiments, previous studies have utilized natural or man-made thermal gradients to examine biological responses to seawater warming over varying timescales [5], [6]. For example, thermal discharge plumes from coastal power plants have provided the opportunity to collect valuable field data to show comprehensive and unexpected changes in marine community structure in response to seawater warming [6], [7]. Longer term warming driven by thermal discharge plumes has also facilitated empirical tests of ecological theories [6] However, while such approaches are clearly valuable, they are inherently either unreplicated or spatio-temporally confounded and also it is not possible for the researcher to exert control of the warming regimes or treatments.

Another common approach that ecologists have adopted examine the influence of environmental drivers on marine biodiversity involves the use of settlement panels, which provide artificial habitat for the settlement and growth of sessile marine organisms. Settlement panels act as uniform, comparable replicates that can be used to conduct manipulative experiments on the influence of key physical and biological processes on community structure. Assemblages colonising settlement panels have been subjected to controlled manipulations of, amongst other factors, physical disturbance, light levels, sedimentation, nutrient enrichment, competition and grazing pressure (e.g., [8], [9], [10], [11]). Here, we developed this approach by manipulating temperature on and around settlement panels to conduct highly novel *in situ* experiments on the response of natural communities to simulated oceanic warming.

## Results

**Figure 1:**
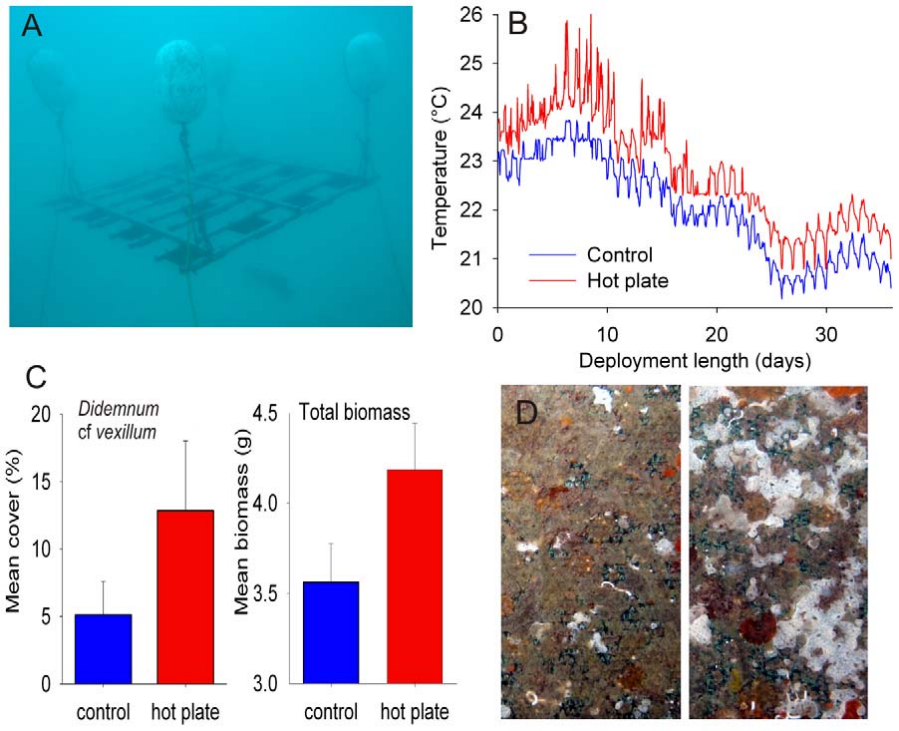
The ‘hot plate’ system was deployed in field (A) and seawater temperature 3 mm over the experimental settling surfaces on both a hot plate and a control plate was recorded for the duration of the deployment (B). Panel C shows mean (± S.E.) percent cover of *Didemnum perlucidum* and total biomass of the sessile assemblage on hot plates and controls (n = 8). Note change of scale on biomass plot, for clarity. A representative sessile assemblage covering a control (left) and hot (right) plate after 36 days is shown in panel D.

A ‘hot-plate’ system, in which electrically heated settlement plates were warmed in an experimental array (comprising 8 hot plates and 8 control plates), was deployed at 6m depth in the fully-saline (35 psu) mouth of the micro-tidal Swan Estuary in Perth, Australia (Fig 1A). The plate surface available for colonisation and the boundary layer of water surrounding the plate were warmed to 0.67°C (±0.11 s.d., based on 860 hourly observations) above ambient temperature, *in situ*, for 36 days (Fig. 1B). The temperature of the Swan River decreased considerably during the deployment, which was conducted in autumn, but the temperature differential between hot plates and control plates remained relatively constant throughout the experiment (Fig. 1B). A typical fouling assemblage, dominated by colonial ascidians, hydroids, bryozoans and barnacles, settled on the plates (Fig. 1D). *Didemnum perlucidum* was the major space occupier; it covered more than twice the space on the hot plates than on the unheated controls (Fig. 1C; t-test on arc-sine transformed data, *df* = 14, t = 1.56, *P* = 0.15). This observation suggested that initial growth and/or development and/or recruitment of *Didemnum perlucidum* may be enhanced by warmer substratum and seawater conditions. Total dry biomass of the fouling assemblage was greater on hot plates than control panels (t-test on Ln transformed data, *df* = 14, t = 1.91, *P* = 0.07), again a likely consequence of accelerated recruitment and/or growth rates of early life stages under warmer conditions.

**Figure 2:**
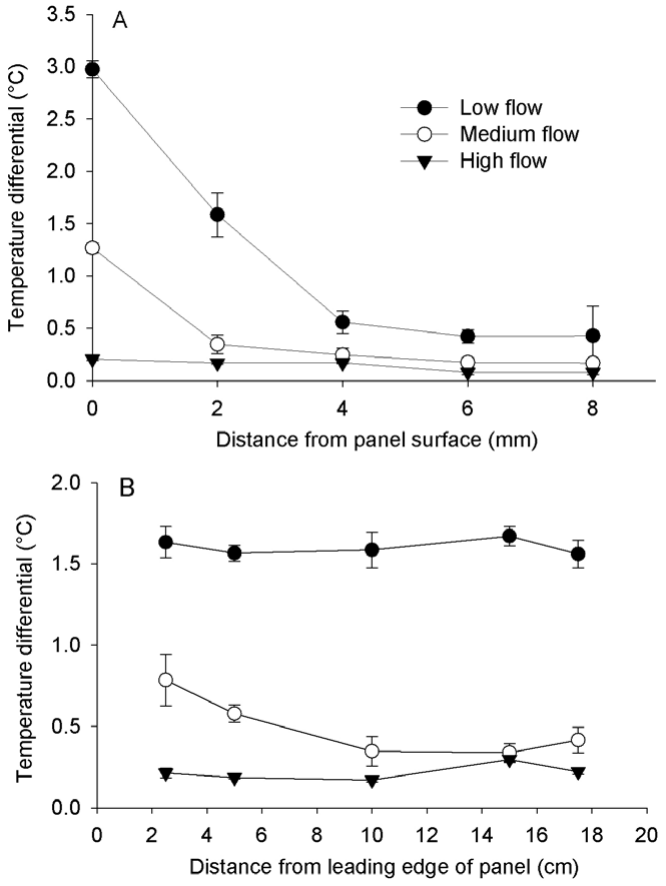
The relationship between distance from the hot plate surface and magnitude of warming under different flow conditions (A). Mean temperature differentials (±SEM) between a control and a hot plate were calculated from 15 readings taken during one minute, following sufficient time for thermal gradients to stabilise. See methods for details of flow conditions. Mean (±SEM) temperature difference between control and hot plate at different positions across the plate surface, under different flow conditions, is shown in panel B.

Further information on the behaviour of the thermal gradient over the hot-plate surface was obtained from an experimental flume system. The relationship between distance from the plate surface and the magnitude of warming was examined under three different flow regimes characteristic of the Swan River. Under low flow conditions, the heated boundary layer of water surrounding the hot plate was in excess of 8 mm in depth, while a maximum temperature differential of 3°C was achieved on the plate's surface (Fig. 2A). However, the thickness of the warmed boundary layer and the magnitude of the warming treatment were strongly influenced by increased flow rates (Fig. 2A). Even so, mean warming of at least 0.2°C was recorded up to 4 mm from the plate's surface at both medium and high current speeds. Crucially, the magnitude of warming across the hot plate surface was consistent under all flow conditions (Fig. 2B).

## Discussion

We have presented a useful technique to modify seawater temperature *in situ*, which is analogous to previous manipulations of environmental factors influencing sessile assemblages on settlement panels. As with other manipulations conducted at small scales, the hot plate system only modifies the immediate environment (i.e. a thin layer of water surrounding the plate surface) and does not elevate the temperature of the water column or the substratum at large spatial scales. As such, the approach has some limitations in that it does not affect larval survivorship, fecundity of the local population, adult physiology, or the pool of species available for colonisation; all of which will be strongly influenced by oceanic warming. The technique is therefore most suited to experimentation on species that have short-lived (or no) life cycle stages in the water column and are primarily influenced by conditions near the substratum, such as microbial biofilms and many species of ascidians and macroalgae. Another limitation relates to the physical extent of the warming treatment, which is (at most) 10 mm in depth and is strongly influenced by water movement across the plate. Consequently, the technique is most appropriate for examining small organisms or life stages that exist within the warmed boundary layer of water, and for experimentation in habitats that experience low to moderate water flow, such as many embayments, lagoons and lakes. Modifications to the panel structure and design to maximise the thickness of the warmed boundary layer are ongoing.

Despite these limitations, the hot plate approach described above has clear benefits. The major advantage of the hot plate system is that it facilitates investigation on the effects of temperature on larval choice, recruitment dynamics, growth and calcification rates, community development and species interactions in real aquatic habitats. This is crucial, as much of the artificiality associated with laboratory, and even mesocosm, approaches is removed by conducting manipulations in real habitats, which are characterised by natural environmental variability and complex interactions that often cannot be simulated under controlled conditions [12]. Manipulations can also be conducted over various timescales, to simulate both gradual warming and short-term extreme events (i.e. heat waves), which are projected to increase in frequency with climate change [1]. Moreover, the system, which can be deployed in any aquatic habitat, can be manipulated like ‘standard’ settlement plates and will thus facilitate complex field experiments that combine multiple stressors (i.e. temperature with disturbance frequency or nutrient enrichment) and examine species interactions (i.e. competition vs. facilitation) within functional communities. Finally, unlike thermal pollution studies, hot plate experiments conducted at different times and places would be comparable, which would permit testing of important climate-related ecological theories, such as the relative effects of warming on organisms inhabiting polar versus tropical systems and thermally stable versus thermally variable environments.

With regards to the biological data from the field deployment, the two key ecological responses to warming we recorded were an increased coverage of the ascidian *Didemnum perlucidum* and a greater total biomass of the sessile assemblage. The genus *Didemnum* includes community-altering invasive species, such as *D. vexillum* and *D. perlucidum*, which have been translocated from their native waters to new habitats on vessel hulls and in ballast waters, sometimes with community-wide consequences [13]. There is some evidence to suggest that the growth and invasive success of these species is correlated with increasing temperatures and reduced water quality [14], [15]. However, very little is known about *Didemnum* spp. in Western Australia and further research is urgently needed. The greater total biomass values we recorded on hot plates compared with controls was most likely due to either increased recruitment or increased growth rates, or a combination of both. Again, more work is needed to investigate the relative importance of these key biological processes in driving this pattern. The potential for oceanic warming to cause an increase in biofouling rates on artificial surfaces has been suggested previously, as microbial biofilm development may accelerate and aggressive fouling seasons may lengthen, particularly in temperate regions [16], [17]. Here, we observed greater fouling on warmer surfaces; an observation that could have major implications for the multi-billion dollar anti-fouling industry.

Most predictions on the consequences of global warming on marine biodiversity have been based either on climate envelope modeling or on inferences from highly artificial laboratory experiments, rather than from observations or manipulations conducted in real habitats. Although these approaches have provided valuable information, they are limited in that they generally do not consider direct or indirect species interactions under the influence of multiple stressors acting simultaneously [18], which even the most sophisticated mesocosms or computer models cannot simulate. Furthermore, most research has targeted individuals or populations, while communities and ecosystems have received far less attention [19]. These pressing knowledge gaps currently restrict our ability to predict, and plan for, the effects of oceanic warming on marine biodiversity and resources [19], [20]. ‘Scaling-up’ results from small-scale experiments to make predictions about large-scale processes such as oceanic warming is challenging [21], and such approaches have been criticised [22]. However, it has been empirically demonstrated that small-scale interactions can generate large-scale patterns [23], while small-scale field experiments have considerably improved understanding of the fundamental principles of ecology [24]. As such, information on the influence of temperature on community structure, species interactions and invasibility gained from field manipulations will enhance our ability to predict the consequences of global environmental change on marine biodiversity and resources. As assigning cause and effect is best achieved through experimental manipulation under realistic conditions [25], the hot-plate system will be a useful addition to the research ‘toolbox’ available to climate scientists in that it will complement modeling, experimental and observational approaches conducted at larger spatial scales.

## Materials and Methods

Hot plates were similar in design to traditional and widely used settlement panels (20×20 cm) except that the surface of the panel and the surrounding boundary layer of water were electrically heated to levels above ambient temperature. The mean magnitude of warming achieved in the Swan River (∼0.7°C) reflected present-day increases above mean temperatures observed during heat waves [26], and the projected increase in mean sea temperature by 2030 [1], [27]. Warming was achieved by mounting an electrical heat trace, sealed in silicon, beneath an anodised aluminium plate. Power was supplied to the heat trace via a 50 m cable that connected to a shore-based control unit and mains power supply. The magnitude of heat transfer through the aluminium plate was a simple function of the applied voltage, which was calibrated prior to deployment. Each panel drew ∼2A current and 25V were applied to the hot plate system throughout the deployment. Therefore, the hot plate system required a maximum power input of 500 watts.

Data on the behaviour of the thermal gradient over the hot plate under different flow conditions were collected from a large experimental flume facility. Unidirectional flow rates were selected to represent typical current speeds observed in the Swan River during tidal cycles: low flow (∼0.01 ms^−1^), medium flow (∼0.05 ms^−1^) and high flow (∼0.12 ms^−1^). Flow was measured with an Acoustic Doppler Velocimeter (ADV); water velocity 4 mm above the hot plate surface was recorded every second for ten minutes (prior to taking temperature readings). Under each flow condition, the temperature of the boundary layer across the hot plate was recorded using high precision T-type temperature probes mounted above the centre of the plate, perpendicular to flow direction. Thermal gradients were allowed to stabilise for 10 minutes, after which temperature was recorded at 0–8 mm distance from the surface of the plate every 4 seconds for a minute. The mean difference (n = 15) between control and hot plate temperatures were then calculated for each minute. Similarly, temperature differentials across the surface of the hot plate (2 mm above the plate's surface and parallel to flow direction), were calculated under the different flow conditions.

For the field deployment, an experimental settling surface (woven shade cloth, 2 mm thick) was fixed to the aluminium plate to serve as substrata for colonisation by marine organisms. Pilot studies showed that the shade cloth facilitated steady, constant diffusion of warm water from the heated metal plate to the surrounding water layer, while providing a suitable surface for rapid colonisation by marine fauna. The array was deployed - with plates facing downwards (to select for fauna rather than flora) and suspended 1 m from the seabed – by scuba divers using an anchor weight at each corner, ropes and buoys. A temperature logger with a needle-pin stainless steel probe was mounted onto a hot plate and a control plate (without altering water motion across the plate surface) to record hourly observations. Following deployment, the shade cloth surfaces were removed from the hot plate system, and returned to the laboratory. Encrusting fauna was identified to the lowest taxonomic level possible and percent cover of each taxon and total dry biomass were recorded for each plate.

## References

[1] Solomon S, Qin D, Manning M, Chen Z, Marquis M (2007). Contribution of working group 1 to the fourth assessment report of the IPCC: Cambridge University Press, Cambridge, UK and New York, USA.

[2] Richardson AJ, Poloczanska ES (2008). Under-resourced, under threat.. Science.

[3] Walther G-R, Post E, Convey P, Menzel A, Parmesan C (2002). Ecological responses to recent climate change.. Nature.

[4] Convey P, Pugh PJA, Jackson C, Murray AW, Ruhland CT (2002). Response of Antarctic terrestrial microarthropods to long-term climate manipulations.. Ecology.

[5] Wernberg T, Thomsen MS, Tuya F, Kendrick GA, Staehr PA (2010). Decreasing resilience of kelp beds along a latitudinal temperature gradient: potential implications for a warmer future.. Ecol Lett.

[6] Schiel DR, Steinbeck JR, Foster MS (2004). Ten years of induced ocean warming causes comprehensive changes in marine benthic communities.. Ecology.

[7] Keser M, Swenarton JT, Foertch JF (2005). Effects of thermal input and climate change on growth of Ascophyllum nodosum (Fucales, Phaeophyceae) in eastern Long Island Sound (USA).. J Sea Res.

[8] Svensson JR, Lindegarth M, Siccha M, Lenz M, Molis M (2007). Maximum species richness at intermediate frequencies of disturbance: consistency among levels of productivity.. Ecology.

[9] Sugden H, Lenz M, Molis M, Wahl M, Thomason JC (2008). The interaction between nutrient availability and disturbance frequency on the diversity of benthic marine communities on the north-east coast of England.. J Anim Ecol.

[10] Maughan BC (2001). The effects of sedimentation and light on recruitment and development of a temperate, subtidal, epifaunal community.. J Exp Mar Biol Ecol.

[11] Russ GR (1980). Effects of predation by fishes, competition, and structural complexity of the substratum on the establishment of a marine epifaunal community.. J Exp Mar Biol Ecol.

[12] Skelly DK (2002). Experimental venue and estimation of interaction strength.. Ecology.

[13] Lambert GJ (2009). Adventures of a sea squirt sleuth: unraveling the identity of *Didemnum vexillum*, a global ascidian invader.. Aquatic Invasions.

[14] Sorte C, Williams S, Zerebecki R (2010). Ocean warming increases threat of invasive species in a marine fouling community.. Ecology.

[15] Agius BP (2007). Spatial and temporal effects of pre-seeding plates with invasive ascidians: Growth, recruitment and community composition.. J Exp Mar Biol Ecol.

[16] Poloczanska ES, Butler A, Durr S, Thomason JC (2010). Biofouling and climate change..

[17] Rao TS (2010). Comparative effect of temperature on biofilm formation in natural and modified marine environment.. Aquat Ecol.

[18] Hawkins SJ, Moore PJ, Burrows MT, Poloczanska E, Mieszkowska N (2008). Complex interactions in a rapidly changing world: responses of rocky shore communities to recent climate change.. Clim Change Res.

[19] Harley CDG, Randall Hughes A, Hultgren KM, Miner BG, Sorte CJB (2006). The impacts of climate change in coastal marine systems.. Ecol Lett.

[20] Wilson SK, Adjeroud M, Bellwood DR, Berumen ML, Booth D (2010). Crucial knowledge gaps in current understanding of climate change impacts on coral reef fishes.. J Exp Biol.

[21] Schneider DC (2001). The rise of the concept of scale in ecology.. Bioscience.

[22] Carpenter SR (1996). Microcosm experiments have limited relevance for community and ecosystem ecology.. Ecology.

[23] Wootton JT (2002). Local interactions predict large-scale pattern in empirically derived cellular automata.. Nature.

[24] Benton TG, Solan M, Travis JMJ, Sait SM (2007). Microcosm experiments can inform global ecological problems.. Trends Ecol Evol.

[25] Underwood AJ (1997). Experiments in ecology: their logical design and interpretation using analysis of variance: Cambridge University Press, Cambridge, UK.

[26] Smale DA, Wernberg T (2009). Satellite-derived SST data as a proxy for water temperature in nearshore benthic ecology.. Mar Ecol Prog Ser.

[27] Poloczanska E, Babcock RC, Butler A, Hobday AJ, Hoegh-Guldberg O (2007). Climate change and Australian marine life.. Oceanograph Mar Biol Ann Rev.

